# Morpho-Physiological Testing of NaCl Sensitivity of Tobacco Plants Overexpressing Choline Oxidase Gene

**DOI:** 10.3390/plants10061102

**Published:** 2021-05-30

**Authors:** Galina N. Raldugina, Sergey V. Evsukov, Liliya R. Bogoutdinova, Alexander A. Gulevich, Ekaterina N. Baranova

**Affiliations:** 1 K. A. Timiryazev Institute of Plant Physiology, Russian Academy of Sciences, Botanicheskaya Street 35, 127276 Moscow, Russia; evsyukov_2013@mail.ru; 2 Plant Cell Biology Laboratory, All-Russia Research Institute of Agricultural Biotechnology, Russian Academy of Sciences, Timiryazevskaya Street 42, 127550 Moscow, Russia; bogoutdinova_lr@rambler.ru; 3 Plant Cell Engineering Laboratory, All-Russia Research Institute of Agricultural Biotechnology, Timiryazevskaya 42, 127550 Moscow, Russia; 4 N.V. Tsitsin Main Botanical Garden of Russian Academy of Sciences, 127276 Moscow, Russia

**Keywords:** salinity, glycine betaine, *Nicotiana tabacum* L., *codA* gene, transgenic plants, osmolytes, proline, salt tolerance

## Abstract

In this study the transgenic lines (TLs) of tobacco (*Nicotiana* *tabacum* L.), which overexpress the heterologous gene encoding the bacterial enzyme choline oxidase were evaluated. The goal of our work is to study the effect of choline oxidase gene expression on the sensitivity of plant tissues to the action of NaCl. The regenerative capacity, rhizogenesis, the amount of photosynthetic pigments and osmotically active compounds (proline and glycine betaine) were assessed by in vitro cell culture methods using biochemical and morphological parameters. Transgenic lines with confirmed expression were characterized by high regeneration capacity from callus in the presence of 200 mmol NaCl, partial retention of viability at 400 mmol NaCl. These data correlated with the implicit response of regenerants and whole plants to the harmful effects of salinity. They turned out to be less sensitive to the presence of 200 mmol NaCl in the cultivation medium, in contrast to the WT plants.

## 1. Introduction

One of the “strategies” for adapting plants to adverse environmental conditions is the accumulation of “compatible” osmolytes that are non-toxic at high concentrations [1]. Compatible osmolytes (osmoprotectants) are water-soluble, low molecular weight organic compounds that ensure stability of the metabolic processes when an organism is exposed to stress factors [2]. One of the most effective osmoprotectors is glycine betaine (GB), a quaternary ammonium compound. The structural features of the GB allow this molecule to interact with both hydrophobic and hydrophilic domains of macromolecules without disrupting cellular functions [3]. GB is known to protect cells from stress by maintaining the osmotic balance between the intracellular and extracellular environment [4], stabilizing the quaternary structure of proteins such as antioxidant enzymes and membrane proteins, as well as multi-protein functional units such as oxygen-secreting complex PS II [5]. However, little is known regarding the mechanism of GB functioning that underlies the plant salt tolerance. Wheat, spinach, beets, and some other crop species are able to accumulate GB in vivo, but levels of this accumulation are too low to adequately control osmotic pressure under stress. However, transgenic wheat expressing the betaine aldehyde dehydrogenase gene is capable of excessive accumulation of GB [6]. Transgenic plants overexpressing GB accumulate this osmolyte at larger quantities than native GB accumulators and show increased resistance to various abiotic stresses [6,7] and have shown that accumulated GB increased the salinity resistance of transgenic wheat overproducing this osmolyte by improving the regulation of osmotic pressure and maintaining ionic homeostasis. Due to the fact that the main and most affordable substrate for the production of GB in plant cells is localized in plastids choline [8], genetic constructs were created to target the synthesized enzyme to plastids [9,10].

One of the attractive enzymes responsible for the synthesis of GB is choline oxidase (E.C. 1.1.3.17) that is found in some bacteria. The enzyme catalyzes the two-step, flavin-linked oxidation of choline to GB, with betaine aldehyde as intermediate and molecular oxygen as electron acceptor [11]. Choline oxidase gene (*codA*) from soil bacterium *Arthrobacter globiformis* has been cloned, characterized, and used in a number of studies to obtain transgenic plants more tolerant to various environmental factors [12,13,14]. A nucleotide sequence of *codA* is saturated by a very high G + C content and can cause a reduction of its activity in subsequent seed generations due to methylation. So, the genetic construct semi-synthetic *codA* gene has been created for improved expression in plants [15].

The aim of this work was to test the hypothesis of the possibility of assessing the salt tolerance of the genotype overexpressing the choline oxidase gene and responsible for the short pathway of GB synthesis at the stage of regeneration. For this, we evaluated the correlation of the regeneration potential with the classical indicators of sensitivity to the toxic effect of NaCl: the content of proline, photosynthetic pigments, and rhizogenesis.

## 2. Materials and Methods

### 2.1. Plant Material

The study used tobacco plants (*Nicotiana tabacum* L.) cv. Samsun. Segments of leaves of aseptic plants propagated by cuttings and grown in vitro for 10–12 weeks were used as explants. The leaf blade with the main vein removed was cut into 0.5–0.7 cm^2^ segments. The culture of aseptic plants was maintained by cutting plants on 0.7% agar Murashige-Skoog (MS) medium for propagation with a half composition of macrosalts and a full composition of microsalts and iron chelate, 0.1 mg L^−1^ α-naphthylacetic acid (NAA), sucrose 0.7% (medium Tp). The plant culture was maintained in a phytotron chamber with a cycle of 12/12 h (day/night) at an illumination intensity of 250 μmol m^−2^ s^−1^ and day/night temperatures of 24–25/19–20 °C.

Seeds for testing transgenicity of plants of the next generations were sterilized for 1 min in 70% ethanol and 20 min in 20% solution of industrial sodium hypochlorite (Domestos, Russia), washed five times with sterile distilled water and then placed on agar (0.7% agar) 1/2 MS medium containing no growth regulators with the addition of 0.5% sucrose and 100 mg L^−1^ of the antibiotic kanamycin (Km) (medium Ts). Petri dishes with seeds were placed in a dark chamber at a temperature of 25 °C and kept for 24 h, then transferred to the phytotron light chamber. Seedlings that remained green were transplanted onto M3 medium with the same Km concentration.

### 2.2. In Vitro Cultivation, Agrobacterial Transformation, and Plant Regeneration

**Figure 1:**
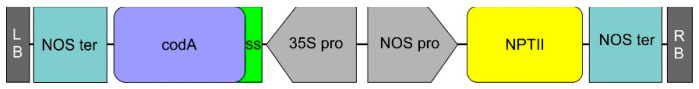
The choline oxidase expression cassette. *codA*—choline oxidase gene with transit peptide ss; NPTII—neomycin phosphotransferase gene; NOS pro—nopalin synthase promoter; NOS ter—nopaline synthase terminator; 35S pro—promoter of cauliflower mosaic virus (CaMV) 35S RNA; RB and LB—right and left border sequences of T-DNA.

Agrobacterium tumefaciens strain AGL0 for the plant transformation was used, which carried a genetic construct based on plasmid pBIcodA with a selectable marker gene *nptII* and with a target gene *codA* (Figure S1a), and which was supplied with a signal sequence from the gene of the small subunit of ribulose bisphosphate carboxylase of tomato that targets the gene product into chloroplast (Figure 1, Figure S1a,b) [10].

Cultures of *A. tumefaciens* were grown in liquid LB medium containing 50 mg L^−1^ rifampicin (Rf) and 50 mg L^−1^ Km with vigorous shaking at 25 °C for 24 h. The explants were transformed by the method of co-cultivation of leaf explants with *A. tumefaciens* cells on the surface of an agar MS medium. The method for obtaining transgenic plants by co-cultivation was described earlier [16]. After 2 days of cultivation on callus-induced medium (Tc) (MS medium containing 3% sucrose, 2 mg L^−1^ α-naphthylacetic acid (NAA), 4 mg L^−1^ kinetin, 0.1 mg L^−1^ 2,4-dichlorophenoxyacetic acid) (2,4-D) in the dark, the explants were transferred to a medium for morphogenesis (Tm) (MS medium containing 1% sucrose, supplemented with 800 mg L^−1^ cefotaxime (Cf) and 100 mg L^−1^ Km). Petri dishes with explants were placed in a light chamber (light cycle 12/12 h day/night at an illumination intensity of 250 μmol m^−2^ s^−1^, day/night temperature was 24–25/20–19 °C). At the end of the two-week incubation, the explants were transferred to the same medium for morphogenesis, but with the addition of 500 mg L^−1^ Cf and 100 mg L^−1^ Km. After 4–5 weeks, the first morphogenic formations appeared on the explants. After 8–10 weeks, the formed shoots were separated from the explants and placed on Tc medium (0.7% sucrose, 1/2 macro-MS, a complete set of trace elements, CaCl_2_ and iron chelate, 0.1 mg L^−1^ NAA). At these stages, Cf was added to this medium at a concentration of 300 mg L^−1^. The Km concentration remained constant. After rooting of shoots and formation of 2–3 leaves, each plant was checked by PCR for the presence of transgenes and expression of the target *codA* gene. Plants containing the *codA* gene were propagated by cuttings and maintained in aseptic culture on Tc medium.

### 2.3. Segregation Analysis

In order to determine the genetic segregation in transformed plants for transgenes, rooted plants were removed from the agar medium and transplanted into vessels with soil (universal soil with the addition of 0.25 volume of perlite) under growth chamber conditions (day/night 12/12, 23–25 °C). The transplanted plants were covered with a plastic bag to preserve turgor. The bag was gradually opened and completely removed after 2–3 days. Seeds were formed in the resulting flowers after self-pollination, which were collected and sown, as indicated earlier, on medium Ts and counted the number of green and white seedlings.

### 2.4. Determination of Salt Stress Sensitivity

To test the sensitivity to salt stress, plants of several lines were propagated by cuttings and planted on Tc medium containing NaCl in a concentration of 0, 200, and 400 mmol and incubated at 25 °C in a growth chamber. After 24 days, the size of the roots was compared for the established rooted plants. Plants with more powerful root systems were selected for further study.

After cuttings, the plants of the selected lines were planted on a fresh medium with salt concentrations of 0 and 200 mM and after 24 days. After growth, sheet material was collected from them, in which the content of glycine-betaine, proline, and pigments were measured.

Moreover, leaf explants were taken from the selected plant lines and after 2 days of incubation on Tc medium, they were divided into two parts, and one part was transferred to a medium for morphogenesis (Tm) without salt or with the addition of 200 mmol or 400 mmol NaCl in medium. The second part was transferred to fresh Tc medium with the addition of NaCl at a concentration of 0, 200, or 400 mmol. After 24 days on the Tm medium, the number of explants with morphogenic formations was counted, and on the Tc medium, the biomass of five formed calli was determined.

### 2.5. Biochemical Analyses

Tobacco regenerants grown in Tp medium in vitro or with the addition of 200 mmol NaCl for four weeks were used. Measurement of GB was performed according to [17]. Fresh leaves (1 g) were mechanically shaken with 20 mL of deionized water for 48 h at 25 °C. The samples were filtered and the filtrates were diluted (1:1) with 2N H_2_SO_4_. Aliquots (0.5 mL) were transferred to centrifuge tubes and incubated in ice water for 1 h. This was followed by adding 2 mL of cold potassium iodide-iodine reagent, gently mixed, and the tubes were stored at 48 °C for 16 h. After centrifugation at 10,000 g for 15 min at 8 °C, the supernatant was carefully aspirated with a 1-mL micropipette. The per iodide crystals were dissolved in 9.0 mL of 1,2-dichloroethane, and the absorbance was recorded spectro-metrically at a wavelength of 365 nm. The reference standards of GB (50–200 mg mL^−1^) were prepared using 1N H_2_SO_4_ [18].

The determination of the content of free Pro was carried out according to the method of [19] with acidic ninhydrin reagent. Extraction of Pro from plant tissue was carried out by boiling a 200 mg sample in distilled water, and then a ninhydrin reagent was added. The color intensity was determined by Specol-11 spectrophotometer (Carl Zeiss, Oberkochen, Germany) at a wavelength of 520 nm against a sample in which distilled water was added instead of the extract. The Pro content was determined from a calibration curve using Serva Pro for its construction. The Pro content was expressed in µmol g^−1^ of fresh weight.

The pigment content was determined by the Shlyk method [20], by extracting pigments from leaves with 96% ethyl alcohol. The degree of absorption of the solution (optical density) for chlorophyll a and b was determined at a wavelength of 665 and 649 nm on a Genesys 20 spectrophotometer (ThermoScientific, Waltham, MA, USA). The pigment concentration was calculated by the formula:

C_chl a_ = 13.70 D_665_−5.76 D_649_; C_chl b_ = 25.80 D_649_−7.60 D_665_ (1)

where C—concentration of pigments, D—optical density. The pigment content is indicated in μg g^−1^ fresh weight.

### 2.6. Molecular Analysis of Regenerants

PCR analysis was carried out in a Tertsik amplifier (DNA Technology, Moscow, Russia) using the following primers to detect the *codA* gene in regenerated plants: *codAf* and *codAr* (Table 1). Amplicon size was 506 bp. Primers for actin: *actin f* and *actin r*. The amplicon size was 496 bp. PCR for both genes was performed in a volume of 25 μL with the following parameters: 2 min at 94 °C, then 30 PCR cycles consisting of 1 min of denaturation at 92 °C, 1 min of annealing at 68 °C, and 1 min of synthesis at 70 °C, then 10 min at 70 °C. Primers for *nptII* gene—*nptIIf* and *nptIIr* were used in the following amplification protocol: 94 °C—4 min, then 30 amplification cycles (94 °C—60 s, 64 °C—60 s, 72 °C—60 s) and the last stage of synthesis at 72 °C for 4 min. All primers were designed with Oligo 7 software (https://www.oligo.net/).

Total plant RNA was isolated by the trizol method. Plant tissue was homogenized with liquid nitrogen. RNA was purified from DNA impurities by treatment with DNAse. The RNA concentration was measured on a Specol-11 spectrophotometer (Carl Zeiss, Oberkochen, Germany) at a wavelength of 260 nm. The reverse transcription reaction was carried out according to the directions of the manufacturer (Fermentas, Lietuvania, Vilnius). The amplified fragments were separated on a 0.8% non-denaturing agarose gel containing ethidium bromide. All primers were designed with Oligo 7 software.

### 2.7. Statistical Data Processing

All experiments were carried out in three biological replicates. The results were statistically processed and expressed as the mean and the representativeness error of the standard deviation. The experimental results were statistically processed using AGROS program (version 2.11). Experimental groups with insignificant differences between mean values according to Duncan’s test (*p* ≤ 0.05) are indicated with the same letters.

## 3. Results

### 3.1. Agrobacterial Transformation and Transformation Efficiency Assessment

**Figure 2:**
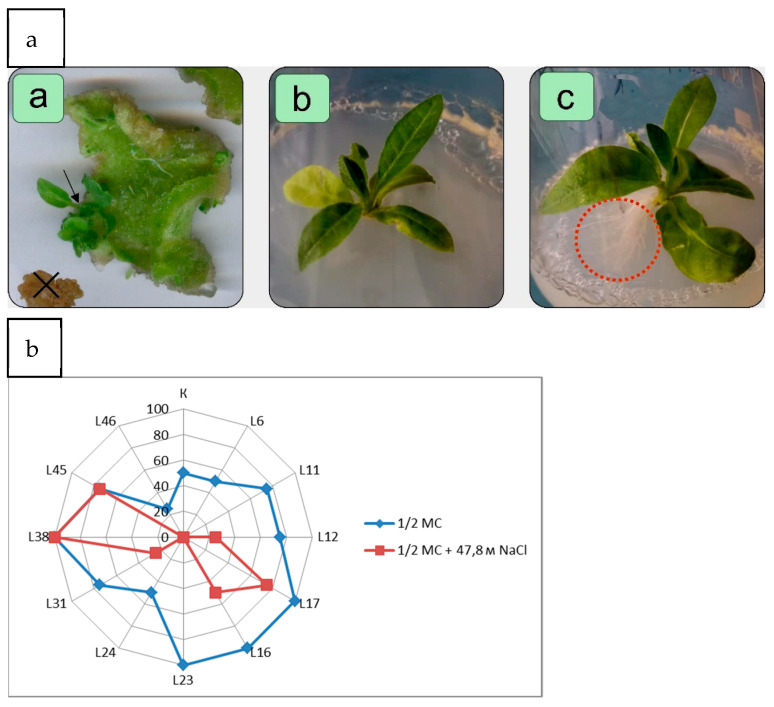
Effect of increased NaCl content on the growth of in vitro plant regenerates: (**a**) Regeneration of shoots and growth individual of the shoots of tobacco (a—shoot formation on leaf explants of tobacco after transformation on medium Tm; b—a shoot of the untransformed line on medium Tp with NaCl; c—a shoot of the transformed line on medium Tp with NaCl). (**b**) The efficiency of rhizogenesis on medium Tp supplemented with 100 mg L^−1^ Km (% root formation). Symbols: arrow—emerging shoot; X—died explant; red circle—regenerant rooting; blue line—Tp; red line—Tp + 50mM NaCl.

The shoots obtained after transformation, which remained green on the medium with Km, were propagated by cuttings and the cuttings were planted for primary screening on the medium Tp, on the same medium, but with the addition of 50 mM NaCl. After 24 days, it was noted that the formation of roots in some of the plants kept in the medium with salt is the same in the media of both variants, while in other lines it is very weak or does not form at all. Figure 2 shows that the putative transgenic lines developed a stronger rooting system in the medium supplemented by NaCl, while the regenerated non-transgenic control line (WT plants) had a weaker rooting system. These results showed that transgenic *codA* plants exhibited greater resistance to salt stress than WT plants.

### 3.2. Screening of Regenerants for the Integration of Heterologous codA Gene Expression

Shoots that formed roots in a medium with a high salt content were tested for transgene by PCR with primers to *codA* (Figure 2). In addition, with DNA isolated from plants in which the presence of the *codA* gene was shown, to exclude the contamination of *A. tumefaciens*, PCR was performed with primers for the *virD2F* gene (results are not shown, since none of the reactions showed the presence of this gene).

**Figure 3:**
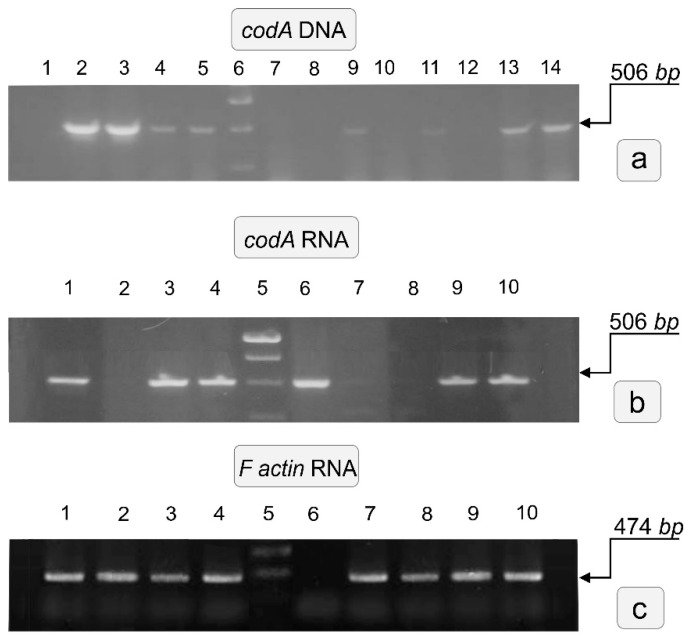
Detection of the *codA* gene integration in regenerated plants. (**a**) Genomic DNA PCR analysis of the *codA* from transgenic plants. Numbers: 1—H_2_O; 2—pBIcodA plasmid; 3—DNA 16 line; 4—DNA 7 line; 5—DNA 17 line; 6—M size marker (10000); 7—DNA of WT; 8—DNA 14 line; 9—DNA 77 line; 10—DNA 31 line; 11—DNA 45 line; 12—DNA 8 line; 13—DNA 38 line; 14—DNA 76 line; (**b**) Detection of the *codA* gene expression by RT-PCR in transgenic lines. (**c**) Detection of the actin gene expression by RT-PCR in transgenic lines. Numbers b and c: 1—line 45; 2—line 31; 3—line 17; 4—line 16; 5—M size marker (10000); 6—pBIcodA plasmid; 7—line 8; 8—line 14; 9—line 8; 10—line 38. The sequence of the tobacco gene was used as an internal standard.

Based on the results of PCR testing of transformed plants, several plant lines were selected, with which further work on the study of their properties was continued. These are the lines: TL16, TL45, TL38, TL17, and TL31. The DNA of these lines was used for PCR reactions with primers to the *virD2F* and *nptII* genes. All reactions with virD2F primers were negative; there was no contamination of plant tissue with agrobacteria. The reaction with primers to the *nptII* gene was positive in all cases (data not shown). So, in most plants, the transgene was present as a complete copy of the introduced genetic construct, and only one of the selected plants (TL31) lacked the target gene. In further experiments, this line was used in some cases as a vector control. It was shown that the *codA* transgene is expressed in all these plants when verifying in these lines by RT-PCR expression at the mRNA level (Figure 3).

### 3.3. Evaluation of the Regeneration Potential of codA Calli of Transgenic Lines during Selection for NaCl

**Figure 4:**
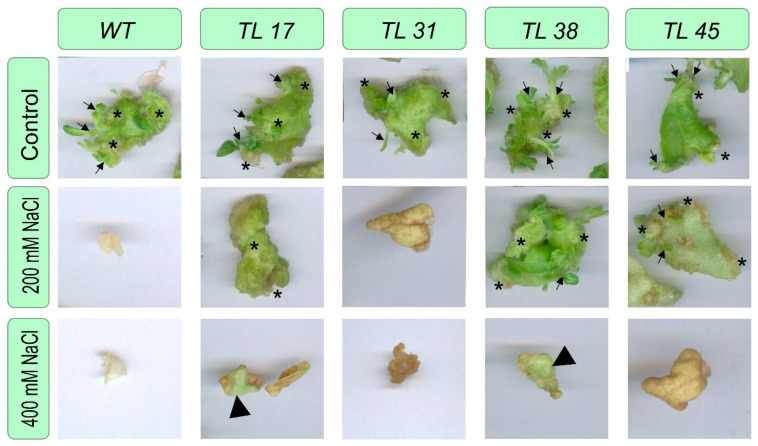
Determination of the in vitro morphogenic and callus forming ability of *codA* transgenic plants on Tm medium with an increased NaCl concentrations: WT—untransformed tobacco plants; TL17, TL38, TL45—*codA* independent transgenic lines. Legend: asterisk—callus; thin arrow—regenerant; large triangle—preservation of viable tissue with partial death cells in the explants.

Plants of these lines were also tested for salt tolerance using an accelerated assay (see Materials and Methods) on a medium containing increased concentrations of NaCl. Leaf explants of several lines were placed on Tm medium containing NaCl at concentrations of 0, 200, and 400 mmol. Explants with morphogenic formations were observed after 24 days (Figure 4).

The results presented in Figure 4 show that after 24 days of incubation of explants on a morphogenic medium, explants of wild-type plants died even at a salt concentration in the medium of 200 mmol, while shoots were formed on explants of transgenic plants. Although, at this concentration, shoot formation proceeded somewhat slower compared to the explants located on a medium without NaCl addition. The most resistant, apparently, is the TL38 line, in which the explants retained the ability to morphogenesis even on a medium containing 400 mmol NaCl, on which the explants had separate morphogenic regions.

**Figure 5:**
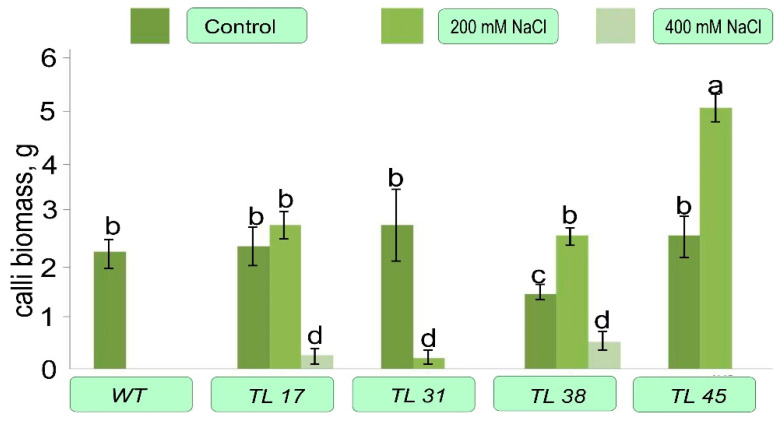
Callus formation ability in *codA* transgenic tobacco lines. The explants were cultured for 24 days on the Tc medium in the dark with a constant temperature of 25 °C and then 5 calli were weighed from each of the Petri dishes. Control medium Tc without the addition of increased concentrations of NaCl, 200—Tc medium with the addition of 200 mM NaCl, 400—Tc medium supplemented with 400 mM NaCl. Columns with the same letters are not significantly different (*p* ≤ 0.05, Duncan’s test).

When comparing the ability of callus formation of the explants, when cultivated on Tc medium, it was shown that explants from non-transformed tobacco plants, as well as on a morphogenic medium, died with increasing salt content in the medium, while explants from transgenic plants showed an increase in callus formation (Figure 5).

The explants from the TL31 had the same performance as the untransformed plant. The most active callus formation was observed in explants from the TL38, where the increase in biomass was almost 100%. Calli on explants from line 45 (TL45) also actively grew. Thus, the callus biomass of TL45 under 200 mmol NaCl was two times higher than the one during growth in the absence of NaCl exposure. But calluses from TL17 grew about the same regardless of the salinity of the medium.

Therefore, from the results of these two tests, it can be concluded that transgenic plants are more resistant to salt. It is likely that this is due to the accumulation of GB synthesized in transgenic plants containing the gene for choline oxidase.

### 3.4. Biochemical Features of Transformed Plants

**Figure 6:**
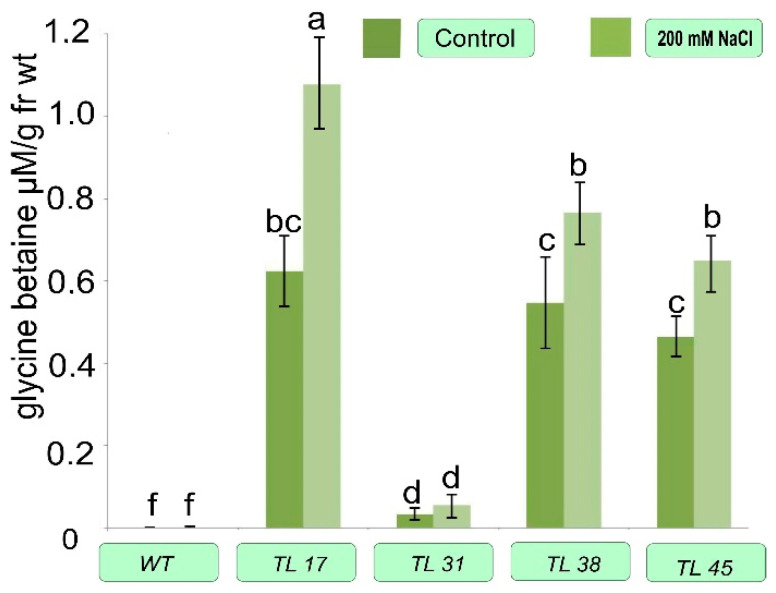
Glycine betaine content in transgenic plant lines. Control—medium Tc without adding NaCl; NaCl—medium Tc with the addition of 200 mmol NaCl. Columns with the same letters are not significantly different (*p* ≤ 0.05, Duncan’s test).

**Figure 7:**
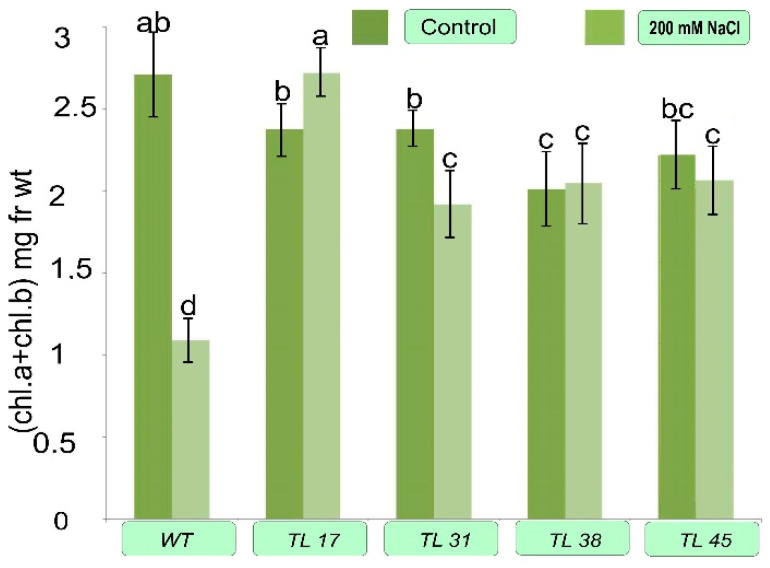
Total chlorophyll content in transgenic plant lines. Control—medium Tc without adding NaCl; NaCl—medium Tc with the addition of 200 mmol NaCl. Columns with the same letters are not significantly different (*p* ≤ 0.05, Duncan’s test).

**Figure 8:**
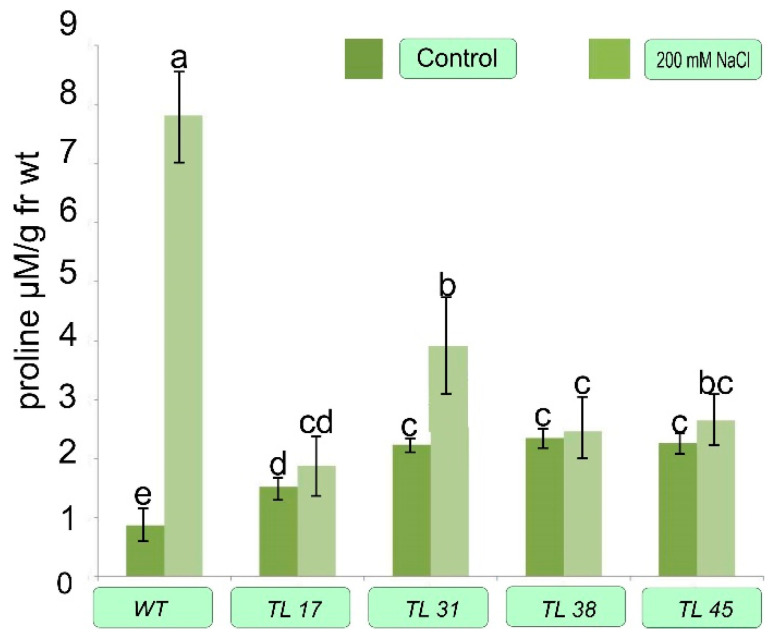
Proline content in transgenic plant lines. Control—medium Tc without adding NaCl; NaCl—medium Tc with the addition of 200 mmol NaCl. Columns with the same letters are not significantly different (*p* ≤ 0.05, Duncan’s test).

Plants of several selected lines were cultured for 24 days in vitro in Tp medium supplemented with 200 mmol NaCl. Then the content of chlorophylls, proline, and GB was determined in their leaves (Figure 6, Figure 7 and Figure 8).

From the results presented in the histogram (Figure 6), data on the increased content of GB in samples of leaves of transgenic plants are presented. In WT plants and TL31, which did not contain the choline oxidase enzyme, the content was insignificant. The stress effect of the increased NaCl content in the cultivation medium in the transformed plants caused an additional increase in the GB content. It is known that GB effectively counteracts the damaging osmotic stress during salinity due to the protective properties of many plants in arid climates [21]. Similar results have been repeatedly demonstrated when using betaine aldehyde dehydrogenase [22], choline oxidase on potato [23,24], tomato [10,24], eucalyptus [25], and tobacco [26,27] plants.

By contrast, WT plants did not accumulate GB either in leaves or in chloroplasts (Figure 6). These values were consistent with the result of Park et al. (2007) in *codA* transgenic tomato of another variety, which revealed the GB content at levels of 9 nmol mg^−1^ chlorophyll [28].

Transgenic plants of lines TL17, TL38, and TL45 accumulated GB in leaves at levels of 0.61, 0.54, and 0.49 nmol g^−1^ fresh weight, respectively (Figure 6), maintaining approximately the same level when exposed to salt. Different genotypes showed small variations in GB levels in response to salt stress in an in vitro experiment. As expected, wild-type plants did not contain GB at all in the leaves and did not respond to stress.

Thus, the steady content of chlorophyll and proline, which remains unchanged under the action of increased salt concentrations that induce the stress, indicates the accumulation of some other compound with a protective effect. Such a compound, apparently, is GB, which is synthesized in our transgenic plants due to the functioning of the introduced choline oxidase gene.

It was shown that the chlorophyll content in the control untransformed plants decreased with increasing salinity from 2.71 µg to 1.09 µg g^−1^ fresh weight (FW). However, it remained almost unchanged, remaining either at the same level or slightly increasing (for TL17, a significant increase of 14%; TL38 and TL45 at the same level). In plants of line 31, which do not have the *codA* gene, the total chlorophyll content decreased by about 20% (Figure 7).

When testing the biochemical properties, in particular of the chlorophyll content, in plants of selected lines cultivated for 24 days on media with different amounts of NaCl, it was shown that the content of chlorophyll (Figure 7) in untransformed plants under the influence of salt decreases by almost 60%, whereas it almost does not change under the action of salt (for TL31, a decrease by 20%, TL17—an increase by 14%, TL38 and TL45—remains the same) for transgenic plants.

When determining the content of proline in these plants from the results presented in Figure 8, it can be seen that in untransformed plants without stress, the proline level is approximately two times lower than in transgenic plants and even in plants of the line where the choline oxidase gene is absent (TL31). When 200 mmol NaCl is added to the medium, the proline level in untransformed plants increases by a factor of 9 (from 0.86 to 7.81 µmol g^−1^ g of FW). Transformed plants of the TL31 line, which have only the selective *nptII* gene, behaved similarly. The proline content in them also increased under the influence of salinity, but in smaller sizes than in untransformed plants (from 1.08 to 2.88 µmol g^−1^ of FW). In transformed tobacco lines which are exposed to salt condition, it remained approximately at the same level, varying within the error.

## 4. Discussion

Salinity caused osmotic and oxidative stress and ionic toxicity. The stability pigment content is considered one of the parameters of salt tolerance of agricultural plants [29]. However, in contrast to the assessment of osmolyte production for the assessment of primary transformants, this method turns out to be ambiguous.

Salt stress is one of the important abiotic stresses that affects plant growth, photosynthesis, and chlorophyll content, the accumulation of osmoprotective solutions, as well as the morphogenic and callus-forming ability of explants obtained from these plants. The stressful impact of the environment on plant cells can lead to a dramatic deterioration in their condition, which at the same time causes adaptation processes in plants. During adaptation, the plant can develop resistance to stress due to the accumulation of some small organic solutes, known as compatible osmoprotective agents or osmoprotectants [30,31]. Osmoprotectors are small neutral molecules that are non-toxic to the cell at molar concentration and stabilize proteins and cell membranes by preventing organelle destruction and denaturing effects of stress on cellular functions [32].

In plants, such compounds include betaine and proline [33,34]. In a number of plant species, regularly exposed to drought and salinization, betaine or glycine, betaine can be synthesized and accumulated in chloroplasts naturally [4]. However, in most plant species, GB is not synthesized in this way. Introducing genes for the synthesis of this compound into plant genomes (by using genetic engineering), it was possible to obtain plants that accumulate GB, which demonstrated greater tolerance to various types of abiotic stress, indicating promising strategies for the development of stress-resistant crops [35,36].

Tobacco plants (*Nicotiana tabacum* L.) are not able to synthesize GB in sufficient quantities and are therefore very sensitive to the stressful influence of high salt concentrations. In the present study, as a result of agrobacterial transformation the green regenerated shoots were obtained from tobacco cv Samsun leaf explants on a selective medium containing the antibiotic Km (Figure 1). After verification by PCR and RT PCR, several plant lines containing and expressing the *codA* transgene were selected (Figure 3) and used in further work. We investigated the effect of NaCl on the resulting transgenic plants, studied in vitro morphogenic and callus-forming ability, as well as some of their biochemical characteristics, manifested under in vitro salt impact.

The obtained transgenic tobacco plants, which contained the gene, encoding for the bacterial choline oxidase enzyme were tolerant without losing the morphogenic and callus-forming ability of leaf explants during salinization of the medium (Figure 4). The *codA* transgenic explants also developed roots well (Figure 2), while the explants of non-transformed plants (WT) and transformed plants containing only the *nptII* gene (TL31) died during growth on medium supplemented with NaCl.

The adverse effect of increased salt concentration on in vitro shoot regeneration and callus formation has also been reported for *Bacopa monnieri* (L.) plants, as well as for *Limnophila aromatic* (Lamk.) Merr [37,38]. These results indicate that salt stress interferes with the regeneration ability of the cells because salt stress affects cell division and elongation, preventing plant growth, decreasing cell numbers, mitotic activity, cell division, and damaging microtubule cytoskeleton [39,40]. Due to the fact that leaf explants from *codA* transgenic tobacco plants grew well under salinity conditions, it can be stated that the synthesized GB reduced the negative effect of salinity, mitigating the osmotic stress induced by NaCl.

Since salinity is known to cause both osmotic stress and ionic toxicity, the chlorophyll content is considered one of the indicators of plant salt tolerance [29]. One of the evidence of transgenic plants tolerance was the chlorophyll content, which changed slightly under the influence of salt. Total chlorophyll remained either at the same level or increased slightly (for TL17, a significant increase of 14%; TL38 and TL45 remained at the same level). In plants of TL31, which lacked the *codA* gene, the total chlorophyll content was reduced by about 20%. In untransformed (WT) plants, the chlorophyll content was decreased by almost 60% under the influence of salt (Figure 7). A decrease in the Chl content under salt stress may be related to destruction of Chl pigments [41]. The loss of Chl during salinization was shown to associate with photoinhibition or the formation of reactive oxygen species [42]. Akcin and Yalcin (2016) demonstrated that plants grown under high salinity conditions have lower stomatal conductance in order to retain water [43]. Consequently, CO_2_ fixation decreases and the rate of photosynthesis declines at such stress. In *codA* transgenic plants, in which the cells synthesize GB, the rate of photosynthesis, apparently, does not change and stomatal conductance can remain at the same level, allowing the chlorophyll content to remain unchanged or slightly increased. This may be due to an increase in the number of chloroplasts in stressed leaves, which was previously noted under salt stress by certain authors in plants of some studied species [44,45].

The accumulation of soluble substances, especially proline, glycine-betaine, and sugars, is a common phenomenon observed under environmental stress [46]. According to many researchers, proline is an important osmolyte for the adaptation of plants to salinity [47,48,49]. It plays a conservative role in the plant tissues as an essential metabolite, and as a signaling molecule under environmental stress [2,46]. Osmotic adjustment by proline protects the cytosol components from dehydration that occurs during salt stress [50]. Proline also has been shown to act as a molecular chaperone, capable of protecting the integrity of membrane proteins and enhancing the activity of various enzymes [46].

Our study of the proline level in WT plants and *codA* transgenic plants under unstressed conditions and under conditions of salt stress showed that the content of proline accumulated in *codA* transgenic plants was much higher than in WT plants (Figure 8). In the absence of stress, the proline level was 0.863 ± 0.058 µmol g^−1^ in WT plants, while in *codA* transgenic plants the proline level was 2.2–2.5 µmol g^−1^. When wild-type plants were subjected to salt stress at 200 mmol NaCl for 24 days, the proline level was 7.8085 ± 0.9751 µmol g^−1^ FW. In contrast, when *codA* transgenic plants were subjected to salt stress at 200 mmol NaCl for 24 days, proline levels were 2.5347 ± 0.7253 and 2.4633 ± 0.04837 µmol g^−1^ FW, significantly less than the response to salt stress in *codA* transgenic rice plants [22]. The absence of a significant increase in proline content in transgenic plants may indicate that salt stress is not a problem for cellular functions. This can be explained by the fact that proline metabolism is regulated either by repression of transcription (or translation) of genes-encoding enzymes, or by feedback inhibition of existing enzymes involved in proline biosynthesis under conditions of salt stress in plants lacking genes for the synthesis of GB, which also accumulates in plants as a versatile tool to cope with environmental stresses such as a salinity.

Like proline, GB imparts stress tolerance. Its role may be to protect the oxygen-releasing PSII complex, stabilize the protein structure of the PSII complex, and maintain ATP synthesis under stress conditions [4,51,52]. It has been shown that GB accumulates in high concentration (4–40 μmol g^‒1^ FW) in naturally accumulating plants such as spinach, sugar beet, and acts as an osmoregulator under conditions of abiotic stress [3].

However, transgenic plants carrying genes of GB biosynthesis produced a much smaller amount of GB: on average, 0.05–5 nmol g^−1^ FW, and only some of them accumulated this osmotic in somewhat larger amounts [4,53]. But even such low GB content in transgenic plants of different species makes them tolerant to salinity or other stresses [26,27,54].

The maintenance of stable photosynthesis and the transport of its products can properly ensure the sustainable growth of the root system and its functioning through the transport of sugars and the maintenance of gravitropism, which is often a target in saline-sensitive plants [55,56,57]. The accompanying processes of division and cell growth are modulation of microtubules while maintaining the corresponding intact dynamics of the cytoskeleton, which is extremely sensitive in plant cells to both osmotic factors and the action of ions [55,58]. The presence of GB appears to prevent damage, water loss, and support the growth of both non-differentiated and differentiated cells in transgenic lines.

So, GB turns out to be an incompletely understood but extremely effective strategy for protecting plants from salinity [59]. Its application on critical crops as wheat, maize, and others can reduce global risks such as warming, desertification, and agricultural salinization [60,61,62].

In our study, a fairly low accumulation of GB was also observed, 0.5–0.6 nmol g^‒1^ FW (Figure 6). Moreover, in vitro genotypes showed small variations in GB content in response to salt stress. In contrast, WT plants did not accumulate GB in the leaves at all, and not respond to stress.

Thus, the concentration of chlorophyll and proline that remains unchanged under the action of increased salt concentrations causing stressful effects, indicates the accumulation of some other compound with a protective effect, which, apparently, is GB, synthesized due to choline oxidase, the gene that is present in our transformed plants.

## 5. Conclusions

In the present study, we demonstrate the prognostic possibilities of assessing the salt tolerance of transgenic plants synthesizing an osmotically active substance, GB, in connection with the processes accompanying sensitivity to medium salinity at the stage of rhizogenesis and cultivation in the presence of 200 mmol salt. This may testify in favor of the prospects of cell selection with the use of concentrations lower than sublethal ones both for evaluating breeding lines obtained by methods of classical selection, mutagenesis, chromosomal engineering, and for preliminary screening of genetically modified plants. It can be assumed that detailed studies of plants selected with a preliminary assessment of morphogenesis and regeneration at the stage of primary transformants will significantly speed up the selection of valuable transgenic lines.

## Figures and Tables

**Table 1:** List of primers used for the confirmation of the transgene presence in putative transformants.

Forward Primer		Reverse Primer	
*codAf*	5′-CGCCAACTTCTTCCAGATCAA-3′	*codAr*	5′-GGGTGTTCATGTCGAACG-3′
*actinf*	5′-CCTCCCACATGCTATTCTCC-3′	*actinr*	5′-AGAGCCTCCAATCCAGACAC-3′
*npt* *IIf*	5′-GTGGAGAAGGCTATTCGGCTA-3′	*npt* *IIr*	5′-CCACCATGATATTCGGCAAG-3′

## Data Availability

Data is contained within the article or supplementary material.

## Supplementary Materials

The following are available online at https://www.mdpi.com/article/10.3390/plants10061102/s1, Figure S1: (**a**) schema of genetic construct, (**b**) schema of GB synthesis.
