# Supplementary figures and images for: Morpho-Physiological Testing of NaCl Sensitivity of Tobacco Plants Overexpressing Choline Oxidase Gene
Source: Plants (Basel). 2021 May 30;10(6):1102. doi: 10.3390/plants10061102 (PMC8227115; doi:10.3390/plants10061102)

**a**

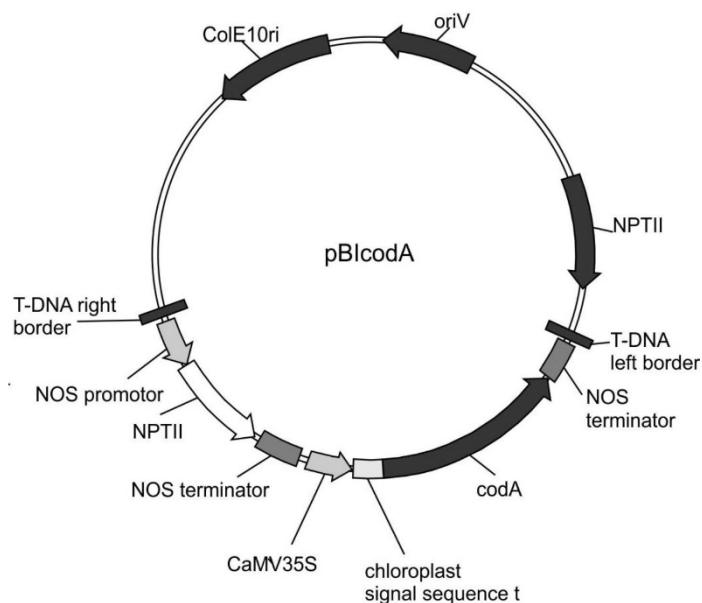

**b**

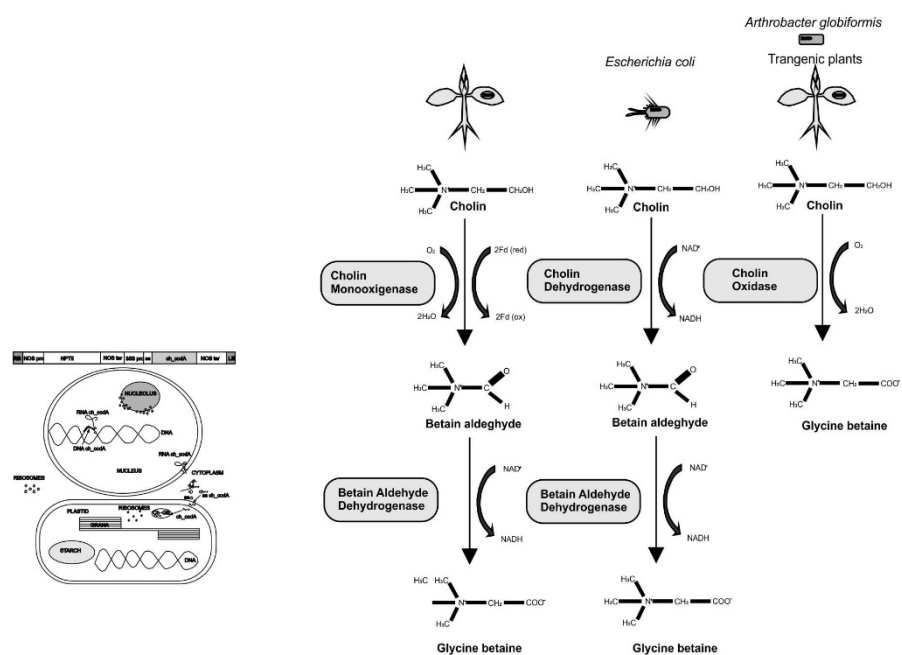

**Figure S1.** (a) schema of genetic construct, (b) schema of GB synthesis.

Supplement: Supplementary file 1 [file plants-10-01102-s001.zip › plants-1210830-supplementary.pdf]
